# Retraction: Adherence of Female Health Care Workers to the Use a Web-Based Tool for Improving and Modifying Lifestyle: Prospective Target Group Pilot Study

**DOI:** 10.2196/101897

**Published:** 2026-06-16

**Authors:** JMIR Publications Editorial Office

**Affiliations:** 1 JMIR Publications Toronto, ON

The JMIR Publications Editorial Office is retracting the article “Adherence of Female Health Care Workers to the Use a Web-Based Tool for Improving and Modifying Lifestyle: Prospective Target Group Pilot Study” [1] due to concerns regarding the integrity of the peer-review process.

Author David Stubljar responded, indicating that there were undisclosed conflicts of interest with two peer reviewers relating to previous copublications. The Editorial Office has determined that there is sufficient evidence indicating that the peer-review process was compromised to proceed with a retraction.

Author David Stubljar agreed with the decision to retract the article; the other authors did not respond to the decision to retract the article.
